# The association among leukocyte apoptosis, autoantibodies and disease severity in systemic lupus erythematosus

**DOI:** 10.1186/1479-5876-11-261

**Published:** 2013-10-19

**Authors:** Yu-Jih Su, Tien-Tsai Cheng, Chung-Jen Chen, Wen-Chan Chiu, Chung-Yuan Hsu, Wen-Neng Chang, Nai-Wen Tsai, Chia-Te Kung, Hung-Chen Wang, Wei-Che Lin, Chih-Cheng Huang, Ya-Ting Chang, Chih-Min Su, Yi-Fang Chiang, Ben-Chung Cheng, Yu-Jun Lin, Cheng-Hsien Lu

**Affiliations:** 1 Departments of Internal Medicine, Chang Gung Memorial Hospital-Kaohsiung Medical Center, Chang Gung University College of Medicine, Kaohsiung Taiwan; 2 Department of Neurology, Chang Gung Memorial Hospital, 123, Ta Pei Road, Niao Sung Hsiang, Kaohsiung Taiwan; 3 Emergency Medicine, Chang Gung Memorial Hospital-Kaohsiung Medical Center, Chang Gung University College of Medicine, Kaohsiung Taiwan; 4 Neurosurgery, Chang Gung Memorial Hospital-Kaohsiung Medical Center, Chang Gung University College of Medicine, Kaohsiung Taiwan; 5 Radiology, Chang Gung Memorial Hospital-Kaohsiung Medical Center, Chang Gung University College of Medicine, Kaohsiung Taiwan; 6 Department of Biological Science, National Sun Yat-Sen University, Kaohsiung Taiwan

**Keywords:** Autoantibodies, Diseases severity score, Leukocyte apoptosis, Systemic lupus erythematosus

## Abstract

**Background:**

Both apoptosis and autoantibodies are important factors associated with disease activity in the pathogenesis of systemic lupus erythematosus (SLE). This study tested the hypothesis that increased leukocyte apoptosis is associated with elevated levels of autoantibodies and the disease activity of SLE.

**Methods:**

Leukocyte apoptosis was determined by flow cytometry, including annexin V, APO2.7, and 7-amino-actinomycin D (7-AAD) on each subtype of leukocyte in 23 patients with SLE. Leukocyte apoptosis was also evaluated in nine patients with Sjogren’s syndrome (SJS) and in 20 volunteer subjects. Titers of common autoantibodies and the disease activity index (SLEDAI-2 k) of the SLE patients were also determined.

**Results:**

Except for annexin V and APO 2.7 of monocytes and late apoptosis (annexin V + 7-ADD) of lymphocytes, apoptosis in the total and in subsets of leukocytes were significantly higher in SLE patients than in controls (all *p* < 0.05, *post hoc* analysis). The mean percentage of late apoptosis of leukocytes (annexin V + 7-AAD) positively correlated with levels of anti-Ro52/60 (*r* = 0.513, *p* < 0.01), anti-La (*r* = 0.439, *p* = 0.04), and anti-Mi-2 (*r* = 0.492, *p* = 0.02), and inversely correlated with both C3 and C4 levels, although not statistically significant. The percentage of APO2.7 of CD19+ cells positively correlated with SLEDAI-2 K score (*p* = 0.01).

**Conclusions:**

Leukocyte apoptosis is significantly higher in patients with SLE and correlates well with the levels of several autoantibodies. The APO2.7 of B-lymphocyte (CD19+) cells positively correlates with the disease activity of SLE.

## Introduction

Systemic lupus erythematosus (SLE) is a chronic systemic disease affecting mostly women of child-bearing age. It is the prototype of autoimmune diseases because of the variety of proposed pathogenesis. Dozens of autoantibodies are linked with SLE patients, including some that are diagnostic and specific [1-3], such as anti-double stranded deoxyribonucleic acid antibody (a-dsDNA), anti-Smith antibody (a-sm), and anti-ribosomal P (a-rib p). The disease activity of SLE has also been associated with these autoantibodies.

Apoptosis is one of several important mechanisms involved in the pathogenesis of SLE. A previous study has demonstrated that lymphocyte apoptosis is increased and removal of apoptotic cells is impaired in SLE. Furthermore, apoptosis is higher in active SLE compared to inactive SLE [4].

Several lines of evidence demonstrate the close relationship of apoptosis and autoantibodies. Delayed clearance of apoptotic residue, such as histone H3 [5], may result in the activation of antigen-presenting cells and the formation of autoantibodies [6,7]. The antigenicity of autoantibodies is mostly against apoptotic cells, which has been demonstrated by Huggins et al. [8]. Most studies link lymphocyte apoptosis with titers of autoantibodies [9,10], but little is mentioned regarding leukocyte apoptosis. The present study focuses on SLE patients due to this lack of detailed data.

To date, little is known about the relationship among leukocyte apoptosis, levels of autoantibodies, and severity of SLE. With the hypothesis that increased leukocyte apoptosis is associated with elevated levels of autoantibodies and disease activity of SLE, this prospectively-designed study aimed to report on the relationship among percentage of leukocyte apoptosis, levels of autoantibodies, and SLE disease activity.

## Patients and methods

### Study patients

Twenty-three patients with definitive diagnosis of SLE and followed-up at the Rheumatology Out-patient Clinic for more than 6 months were prospectively evaluated. The diagnostic criteria for SLE were based on the 1997 revision of 1982 American College of Rheumatology (ACR) classification criteria for SLE [3], while the clinical assessment of disease activity of SLE was according to the SLE disease activity index (SLEDAI-2 K) [11]. For comparison, 20 age- and sex-matched healthy subjects year were enrolled as healthy controls and nine patients with Sjogren’s syndrome (SJS) were the disease controls. The SJS patients were diagnosed based on classification criteria for SJS [12].

The Institutional Review Committee on Human Research approved the study protocol and the participants provided informed consent. Patients were excluded if they had autoimmune diseases other than SLE or SJS, or if they had fever or any infectious disorder that could affect the WBC count.

### Clinical assessments

All of the subjects underwent complete medical examinations upon enrollment. Biomarkers were also collected. Since there were no clinical practice guidelines for SLE flare-up, the important decision in immuno-suppressant adjuvant treatment was whether or not to add a cytotoxic or corticosteroid-sparing drug like cyclophosphamide, methotrexate, azathioprine, mycophenolate mofetil, or leflunomide. Severe SLE was defined as an SLEDAI-2 K score >12, while mild-to-moderate SLE was an SLEDAI-2 K score ≤12 [13].

### Blood sampling and assessment of leukocyte apoptosis

Blood samples were collected by venipuncture of the patient’s forearm vein.

#### Flow cytometry assay using APO 2.7 Antibody for detecting apoptosis

All of the flow cytometry assays were performed within one hour after blood extraction to ensure that the results were as close as possible to the *in vivo* situation. Fixed amounts of blood were diluted 1:5 with PBS, and 100 μL was stained with 10 μL of each of the following: fluorescence conjugated monoclonal antibodies against CD45-phycoerythrin (PE)-Cy5 (clone J33), CD61-fluorescein isothiocyanate (FITC; clone SZ21), and APO 2.7-PE (clone 2.7A6A3; Immunotech, Marseille, France). These were then titrated at saturating concentrations.

The CD45-PE-Cy5 antibody reacted with the CD45 family of trans-membrane glycoproteins, expressed on the surface of all human leukocytes, and a pan-leukocyte marker. The CD61-FITC antibody was a pan-platelet marker that reacted with the GPIIb/IIIa complex (fibrinogen receptor). The APO 2.7-PE antibody reacted with a 38-kDa mitochondrial membrane protein (7A6 antigen), which was detectable on non-permeabilized cells in the late apoptotic state [14].

Annexin V staining, relevant to early apoptosis, produced similar results but was rejected for questionable reliability under fixation conditions, with formaldehyde clearly biasing the staining results. Mouse immunoglobulin G-PE was a control for non-specific staining, which did not differ from the APO2.7-PE signal on platelets, such that each subject was its own control without changing the sample tube. After 30 min of incubation in the dark at room temperature, the stained samples were diluted with 0.5 ml of FACSFlow (Becton Dickinson, San Jose, CA).

Flow cytometry analysis was performed immediately after staining using an Epics XL flow cytometer (Beckman Coulter, Fullerton, California) and the CellQuest software. Five thousand CD45-PE-Cy5+ cells per sample were acquired in a combined forward and side scatters and deep-red FL4 fluorescence (CD45-PE-Cy5) leukocyte gate. Another 5000 CD61-FITC + cells per sample were acquired in a combined forward and side scatters and green FL1 fluorescence (CD61-FITC) platelet gate to define a negative control threshold for the measurement of apoptosis, such that each subject was its own control.

#### Annexin V-FITC 7-AAD fluorescence-activated cell analysis

Membrane phosphatidyl-serine was detected by annexin-V using a commercially available kit (Boehringer Mannheim, Indianapolis, Indiana). The PBS-washed leucocytes were incubated with annexin V-FITC and 7-amino-actinomycin D (7-AAD) for 15 min at room temperature according to manufacturer’s guidelines. Samples were transferred to 5-mL polypropylene tubes, diluted 900 μL with Hanks’ balanced salt solution, and placed on ice before analysis by flow cytometry. The samples were analyzed using an Epics XL flow cytometer (Beckman Coulter, Fullerton, California) and the CellQuest software. Fifteen thousand events were counted per sample. Low-fluorescence debris was gated out of the analysis. Leukocyte subtypes were identified according to their CD45 expression intensity and divided into neutrophils, monocytes, and lymphocytes. From here on, white blood cells (WBC) represented total leukocytes.

Annexin V-FITC staining was identified in fluorescent-1 and 7-AAD staining in fluorescent-4. Cells were identified as follows: early apoptotic cells if they were positive for marker annexin V-FITC but negative for 7-AAD; late apoptotic cells if they were positive for annexin V-FITC and 7-AAD; dead cells if they were negative for annexin V-FITC but positive for 7-AAD; and viable cells if they were negative for annexin V-FITC and 7-AAD.

#### Apoptosis of lymphocytes

Fixed amounts of blood were diluted 1:5 with PBS and 100 μL was stained with 10 μL of each of the following: fluorescence conjugated monoclonal antibodies against CD4-phycoerythrin (PE)-Cy5, CD19-fluorescein isothiocyanate (FITC), and CD8- phycoerythrin (PE). Each of the above staining was further stained with annexin V-FITC, 7-amino-actinomycin D (7-AAD), or APO 2.7-PE (clone 2.7A6A3; Immunotech, Marseille, France). These were then titrated at saturating concentrations. Samples were transferred to 5-mL polypropylene tubes, diluted with 900 μL of Hanks’ balanced salt solution, and placed on ice before flow cytometry. Samples were analyzed using an Epics XL flow cytometer (Beckman Coulter, Fullerton, California) and the CellQuest software. Fifteen thousand events were counted per sample. Lymphocyte subtypes were identified according to their surface antigen (i.e., CD4, CD8, or CD19) expression intensity. A database coordinator was responsible for monitoring all data collection and entry.

#### Autoantibody detection

Autoantibody titers, including IgG antibodies against dsDNA (a-dsdna), Ribosomal p (a-ribp), Ro (52/60) (a-ro), Ro 52 kDa (a-ro52), Ro 60 kDa (a-ro60), La (a-la), U1RNP (a-u1rnp), Sm (a-sm), and Mi-2 (a-mi2) were detected by Elia-Phadia 250 according to the operation manual. All data were checked for any inconsistencies. Intra-assay variability based on repeated measurements of the same blood sample was low, with mean coefficients of variance between 6.11% and 14.28% for SLE patients, and 6.0% in control individuals.

### Statistical analysis

Data were expressed as mean ± SD or median (inter-quartile range). Categorical variables were compared using Chi-square test or Fisher’s exact test, as appropriate. The percentage of leukocyte apoptosis and autoantibody level were logarithmically transformed to improve normality. Univariate analyses were compared using Student’s t-test. Continuous variables among the three groups (SLE, SJS, and volunteer control) were compared using one-way ANOVA, followed by Bonferroni post hoc multiple comparison procedure. Correlation analysis was used to evaluate the relationship among mean SLEDAI-2 K score, percentage of leukocyte apoptosis, and levels of autoantibodies. Statistical significance was set at *p* < 0.05. Receiver operating characteristic (ROC) curves were generated for leukocyte apoptosis. The areas under the ROC curves (AUC) were calculated for each parameter and compared. All statistical calculations were performed using the SAS software package, version 9.1 (2002, SAS Statistical Institute, North Carolina).

## Results

### Baseline characteristics of the study patients

The baseline characteristics and laboratory data of SLE patients, SJS patients, and control groups, as well as their medications, were listed in Table 1. Although the mean age was older in the SJS group, there was no statistically significant difference in age and sex among the three groups (*p* = 0.16 and *p* = 0.541, respectively). The clinical symptoms of the 23 SLE patients included musculo-skeletal involvement in 15, neurologic involvement in 14, muco-cutaneous involvement in two, hematologic involvement in six, cardio-respiratory involvement in two, and renal involvement in two. The clinical symptoms of the nine patients with SJS were also listed.

**Table 1:** Baseline characteristics of SLE patients, SJS patients, and control subjects

	**SLE patients** **(n = 23)**	**Sjogren's syndrome** **(n = 9)**	**Healthy controls** **(n = 20)**
Age (y) (mean ± SD)	44.38 ± 10.87	56.56 ± 13.86	48.37 ± 12.01
Male /female	2/21	1/8	6/14
Clinical symptoms	^Ϯ^		
Constitutional	9	0	---
Muco-cutaneous involvement	2	0	---
Neurological involvement	14	7	---
Musculo-skeletal involvement	15	2	---
Cardio-respiratory involvement	2	0	---
Renal involvement	3	0	---
Hematologic involvement	6	0	---
Median (IQR) SLEDAI-2 K	11 (6-14)		---
Mean dosage of medication (mg/day)	ξ	^ᶃ^	
Prednisolone	21 (mg/day) (n = 15)	3.3 (mg/day) (n = 5)	---
Hydroxychloroquine	300 (mg/day) (n = 17)	150 (mg/day) (n = 5)	---
Azathioprine	50 (mg/day) (n = 1)	0	---
Mycophenolate	1080 (mg/day) (n = 1)	0	---
Cyclophosphamide	500 (mg/month) (n = 2)	0	---
Cyclosporine	50 (mg/day) (n = 3)	0	---

^Ϯ^17 patients who had more than one clinical symptom.

^ξ^12 patients who took more than one medication.

^ᶃ^4 patients who took more than one medication.

*Abbreviations*: *SLE* Systemic lupus erythematosus, *SJS* Sjogren’s syndrome, *IQR* Inter-quartile range, *SLEDAI-2 K* Systemic lupus erythematosus disease activity index 2000.

### Leukocyte apoptosis in patients with SLE, patients with SJS, and the controls

The laboratory data and percentage of leukocyte apoptosis of the three groups were listed in Table 2. Except for hemoglobin level, the WBC and platelet counts were not significantly different among the three groups. Except for annexin V and APO2.7 in monocytes and annexin V + 7AAD in lymphocytes, the percentages of apoptosis of total leukocytes and the leukocyte subsets were significantly higher in the SLE patients than in the controls.

**Table 2:** Laboratory data of the SLE patients and control subjects

	**SLE patients** **(n = 23)**	**SJS patients** **(n = 9)**	**Healthy controls** **(n = 20)**	***p*value**
White blood cells (×103/ml)	6.73 ± 2.5	6.47 ± 1.97	5.68 ± 1.89	0.442
% granulocyte	69.49 ± 13.19	65.93 ± 10.22	61.6 ± 12.76	0.341
% lymphocyte	22.81 ± 11.65	25.33 ± 9.15	31.01 ± 10.85	0.232
% monocyte	5.57 ± 3.00	6.19 ± 2.44	5.63 ± 1.74	0.873
Hemoglobulin (mg/dL)	11.73 ± 1.73	12.38 ± 0.99	14.06 ± 1.86	0.003^α^
Platelet counts (×104/ml)	22.13 ± 8.49	23.67 ± 5.39	22.26 ± 6.19	0.851
Autoantibodies titers				
a-dsdna	18 (1.7, 70.0)	ND	ND	ND
a-ro	0.5 (0.1, 240)	84.75 (0.3, 217.5)	ND	0.725
a-ro52	0.3 (0.1, 32)	ND	ND	ND
a-ro60	0.9 (0, 300)	ND	ND	ND
a-la	0.2 (0, 3.3)	0.2 (0.1, 4.45)	ND	0.896
a-mi2	0.1 (0, 0.2)	ND	ND	ND
Apoptosis tested by flow cytometry^?^				
Leukocyte apoptosis (%)				
Annexin V (%)	15.3 (8.9-18.2)	9.87 (8.29-12.50)	10.9 (8.8-13.4)	0.005^β^
APO2.7 (%)	1.3 (0.9-2.2)	1.10(0.70-1.57)	0.7 (0.5-0.9)	0.001^γ^
Annexin V + 7AAD (%)	7.1 (3.8-11.8)	6.58(2.80-10.10)	3.2 (2.6-4.5)	0.011^δ^
Neutrophil apoptosis (%)				
Annexin V (%)	21.6 (11.7-28.8)	9.15 (7.80-15.76)	13.9 (10.3-18.0)	0.010^ϵ^
APO2.7 (%)	0.6 (0.4-1.3)	0.79(0.40-1.05)	0.5 (0.4-0.6)	0.001^ζ^
Annexin V + 7AAD (%)	10.7 (3.1-22.8)	6.93(1.97-20.60)	3.9 (2.2-5.5)	0.026^η^
Lymphocyte apoptosis (%)				
Annexin V (%)	8.4 (4.9-12.1)	6.10 (5.11-8.23)	4.8 (3.8-6.7)	0.009^θ^
APO2.7 (%)	0.7 (0.5-1.0)	0.46(0.43-0.78)	0.3 (0.2-0.4)	0.008^κ^
Annexin V + 7AAD (%)	2.1 (1.2-3.6)	1.46(0.67-2.92)	1.5 (0.9-2.1)	0.101
Monocyte apoptosis (%)				
Annexin V (%)	19.6 (13.5-27.3)	13.83 (11.42-15.38)	17.4 (12.4-21.6)	0.032^λ^
APO2.7 (%)	3.0 (2.5-6.8)	1.94 (1.48-3.27)	1.8 (1.1-2.7)	0.088
Annexin V + 7AAD (%)	10.4 (5.3-17.8)	7.63 (3.34-15.36)	4.6 (3.3-6.0)	0.024^μ^

*Abbreviations*: *SLE* Systemic lupus erythematosus, *SJS* Sjogren’s syndrome, *WBC* White blood cells or leukocytes, *7-AAD* 7-amino-actinomycin D, *SD* Standard deviation, *IQR* Inter-quartile range, *A-dsdna* Anti-dsDNA, *A-ro* Anti-Ro52/60, *A-ro52* Anti-Ro 52 kDa, *A-ro60* Anti-Ro 60 kDa, *A-la* Anti-La, *ND* Not done.

^Ϯ^Figures are median (IQR) or number (%).

Results of Bonferroni multiple comparisons for post hoc test (SLE vs. control, SLE vs. SJS, or SJS vs. control).

α, SLE vs. control = 0.002; β, SLE vs. control = 0.014, SLE vs. SJS = 0.029; γ, SLE vs. control = 0.001; δ, SLE vs. control = 0.009; ϵ, SLE vs. control = 0.005, SLE vs. SJS = 0.004; ζ, SLE vs. control = 0.041; η, SLE vs. control = 0.021; θ, SLE vs. control = 0.008; κ, SLE vs. control = 0.007; λ, SLE vs. SJS = 0.036; μ, SLE vs. control = 0.026.

### Correlation of clinical severity, autoantibody level, and percentage of leukocyte apoptosis

Regarding the innate immune cells, the mean percentages of late apoptosis of leukocyte (annexin V-FITC and 7-AAD) positively correlated with levels of anti-dsDNA (*r* = 0.415, *p* < 0.05), anti-Ro52/60 (*r* = 0.513, *p* < 0.01), anti-La (*r* = 0.439, *p* = 0.04), anti-Mi-2 (*r* = 0.492, *p* = 0.02), and inversely correlated with both C3 and C4 levels, although not statistically significant (Table 3). The SLE disease activity index (SLEDAI-2 K) positively correlated with anti-Ro 60 kDa (*r* = 0.407, *p* = 0.05), but negatively correlated with complement C4 level (*r* = -0.508, *p* = 0.01).

**Table 3:** Correlation among apoptotic markers, disease activity, and levels of autoantibodies in lupus patients

		**SLEDAI2k**	**c3**	**c4**	**a-ro**	**a-ro52**	**a-ro60**	**a-la**	**a-mi2**
(7AAD + annexinV) of WBC (%)	r	0.14	-0.3	-0.19	.513*	0.29	0.41	.439*	.492*
	*p* value	0.54	0.17	0.39	0.01	0.17	0.05	0.04	0.02
(7AAD + annexinV) of Granulocyte (%)	r	0.18	-.460^*^	-0.2	0.33	0.2	0.32	0.33	0.3
	*p* value	0.42	0.03	0.37	0.13	0.36	0.13	0.13	0.16
(7AAD + annexinV) of CD8 (+)(%)	r	-0.06	0.35	.636^*^	-0.07	0.07	-0.21	-0.09	-0.11
	*p* value	0.87	0.33	0.04	0.85	0.85	0.57	0.8	0.77
APO2.7 of CD4(+)(%)	r	0.3	0.17	0.05	-.684*	-.717*	-0.62	-0.57	-0.24
	*p* value	0.43	0.67	0.9	0.04	0.03	0.08	0.11	0.54
APO2.7 of CD19 (+)(%)	r	.793*	-0.45	-0.48	0.32	0.34	0.29	0.4	-0.07
	*p* value	0.01	0.22	0.19	0.4	0.37	0.45	0.28	0.86
SLEDAI2k	r	X	-0.35	-.508*	0.3	0.23	.407*	0.24	0.21
	*p* value		0.1	0.01	0.15	0.29	0.05	0.25	0.32

Note: *, p<0.05.

*Abbreviations*: *R* Correlation coefficient, *A-ro* Anti-Ro52/60, *A-ro52* Anti-Ro 52 kDa, *A-ro60* Anti-Ro 60 kDa, *A-la* Anti-La, *A-mi2* Anti-Mi-2, *WBC* White blood cells or leukocytes, *7-AAD* 7-amino-actinomycin D, *SLEDAI-2 K* Systemic lupus erythematosus disease activity index 2000.

Regarding the adaptive immune cells, the percentage of CD4 (+) cell apoptosis in APO2.7 negatively correlated with anti-Ro 52 kDa (*r* = -0.717, *p* = 0.03), and anti-Ro52/60 (*r* = -0.684, *p* = 0.04). The percentage of CD19 (+) cell apoptosis in APO2.7 positive correlated with SLE disease activity index (SLEDAI-2 K) (*r* = 0.793, *p* = 0.01), but negative correlated with a-dsDNA (*r* = -0.883, *p* < 0.001). Furthermore, the AUC for the percentage of APO2.7 in B-lymphocyte (CD19+) apoptosis was 0.95 (*p* = 0.027, 95% CI: 0.00-1.00). The cut-off value of the percentage of APO2.7 in B-lymphocyte (CD19+) apoptosis for severe SLE was 1.01% (sensitivity 75% and specificity 95%). Other results were listed in Table 3.

## Discussion

The present study has several major findings. First, the percentage of leukocyte apoptosis (including neutrophil, monocyte, and lymphocyte apoptosis) is significantly higher in SLE patients than in healthy controls. Second, the mean percentages of late apoptosis of leukocyte (annexin V + 7-AAD) positively correlate with levels of most common autoantibodies (e.g. anti-Ro52/60, anti-La, and anti-Mi-2). Third, the percentage of CD4 (+) cell apoptosis in APO2.7 significantly but negatively correlates with levels of several autoantibodies (e.g. anti-Ro52/60, and anti-Ro52kDa). Fourth, the level of C3 negatively correlates with the late apoptosis of granulocyte (7-AAD + annexin V). The level of C4 positively correlates with the late apoptosis (7-AAD + annexin V) of CD8 cells. Lastly, the percentage of APO2.7 on B-lymphocyte (CD19+) apoptosis significantly correlates with median SLEDAI-2K score. The cut-off value of 1% of APO2.7 on B-lymphocyte (CD19+) apoptosis is associated with severe SLE. APO2.7 is expressed on the mitochondrial membrane during early apoptosis in relation to the release of cytochrome *c* and represents the activation-induced cell death under certain circumstances [15,16].

Several lines of blood cells have defects in the apoptosis pathway in SLE patients, either prone or resistant to apoptosis [17-23]. Some studies reveal increased monocyte and lymphocyte apoptosis [18,20,21,23], but other studies demonstrate increased leukocyte apoptosis [22], and decreased B-lymphocyte apoptosis [17] or no difference in apoptosis [19]. Generally speaking, in the present study, the percentages of apoptosis in most leukocyte subsets, including neutrophils, monocytes, and lymphocytes, are significantly higher in SLE patients than in controls.

Leukocyte late apoptosis markers are positively correlated with levels of several autoantibodies, including anti-Ro, anti-La, and anti-Mi2. This is an evident link between leukocyte apoptosis and antibody formation, as previously proposed [24,25].

The CD4 (+) cell apoptosis marker APO2.7 is negatively correlated with levels of anti-Ro52, and anti-Ro. Since the anti- Ro antibody is elevated in many patients with lupus [26], CD4 (+) cell apoptosis may be decreased in SLE patients. The present study demonstrates that the APO2.7 of CD19+ lymphocytes is positively correlated with disease activity index. Several studies have demonstrated that the appearance of APO2.7 on lymphocytes represents activation and elevated titers of autoantibodies [16,27]. However, activated APO2.7 on lymphocytes may be reversed by downstream anti-apoptosis molecules, as shown in other studies [15,28]. Lastly, increased activated APO2.7 of CD19+ lymphocytes may undergo apoptosis after immuno-suppressant therapy [29]. Thus, in this study, activation through a mitochondria-dependent pathway among CD19+ lymphocytes is prominent in patients with active SLE. Disease activity is positively correlated with anti-Ro60 titers in this study, as previously mentioned in other investigations [1,2].

During SLE flare-up, both C3 and C4 are decreased, so the late apoptosis of granulocyte increases [30] but the apoptosis of CD8 (+) cells decreases. The present study also demonstrates that the level of C3 is significantly but negatively correlated with the late apoptosis of granulocyte (7-AAD + annexin V), while the level of C4 is positively correlated with the late apoptosis (7-AAD + annexin V) of CD8 cells (Table 3). Linker-Israeli et al. have shown that CD8 (+) cells participate in autoantibody synthesis in SLE [31].

There are significant differences of apoptosis markers in annexin V of leukocytes (SLE vs. SJS, *p* = 0.029), neutrophils (SLE vs. SJS, *p* = 0.004), and monocytes (SLE vs. SJS, *p* = 0.036) during the *post hoc* analysis, despite no difference in absolute numbers of leukocytes, neutrophils, and monocytes between SLE and SJS patients. Similarly, Armstrong et al [22] demonstrated higher annexin V of neutrophils among SLE patients compared to those with other autoimmune diseases, while Bengtsson et al [21] demonstrated higher annexin V of monocytes. These finding suggest that there are more autoantigens, which may the induce apoptosis of leukocytes, in SLE patients than in the disease controls.

This study has several limitations. First, there are many assays for evaluating apoptosis *in vitro* so the results and interpretations from various methodologies may be partially different. The level of apoptosis tested by flow cytometry may not necessarily reflect the real leukocyte physiologic function *in vivo*. Similar protocols are used to detect cell apoptosis [29,32,33]. Second, this is a cross-sectional observational study. Theoretically, high-dose immuno-suppressant drugs can affect leukocyte activity and autoantibody levels. Although the dosages of steroids (mean, 31 mg/day) and hydroxychloroquine (mean, 300 mg/day) used in this case control study are not high, their effects still cannot be excluded. Lastly, the case number in this study is not large and similar aspects of results have been obtained from other series studies with small case numbers [34,35]. Large-scale prospective and longitudinal studies are needed to evaluate the prognostic contribution of leukocyte apoptosis on clinical outcome.

In conclusion, leukocyte apoptosis is significantly higher in SLE patients and correlates well with the levels of several autoantibodies. The APO2.7 of B-lymphocyte (CD19+) cells positively correlates with the disease activity of SLE.

## Ethical approval

The study was approved by Chang Gung Memorial Hospital’s Institutional Review Committee on Human Research.

## Competing interests

The authors declare that they have no competing interests.

## Author contributions

YJS participated in the study design and manuscript drafting. TTC, CJC, WCC, CYH, WNC, CTK, HCW, WCL, CCH, YTC, CMS, YFC, BCC, and YJL participated in the sequence alignment and clinical evaluation of patients. NWT performed the statistical analysis. CHL conceived the study, and participated in its design and coordination, and helped draft the manuscript. All authors read and approved the final manuscript.
